# Foreign executives, digital transformation, and innovation performance: Evidence from Chinese-listed firms

**DOI:** 10.1371/journal.pone.0305144

**Published:** 2024-06-07

**Authors:** Dong Shao, Kangyin Lv, Xueyuan Fan, Bochen Zhang

**Affiliations:** 1 Business School, Northeast Normal University, Changchun, China; 2 School of Management, Jilin University, Changchun, China; Shanghai Business School, CHINA

## Abstract

This study investigates the effect of foreign executives on firms’ innovation performance and the mediation role of digital transformation in Chinese-listed firms from 2011 to 2021. Our findings indicate that the presence of foreign executives in top management teams promotes firms’ innovation performance by enhancing digital transformation. Further analyses show that foreign executives contribute significantly to improving firms’ radical innovation performance rather than incremental innovation performance. We also examine the moderating effect of negative performance feedback and financing constraints between foreign executives and innovation performance, finding that foreign executives can promote innovation performance particularly in firms with negative performance feedback and weak financing constraints.

## 1. Introduction

The rapid development of economic globalization and increasing transnational business activities have created new skills and attributes requirements for firms to improve their international business abilities [1]. To quickly acquire advanced management methods and experience from developed countries and expand overseas resource channels and international business, firms have greatly increased their demand for foreign management talent. An increasing number of firms are introducing foreign executives into top management teams (TMTs) at high salaries [2]. The expectation is that foreign TMT members will be unaffected by domestic human interference, rely on their professional experience and overseas advantages, participate in firm strategy formulation, and enhance the competitive advantage of firms [3]. Previous research shows a close connection between foreign executives and firms’ strategic decision making [4, 5]. Compared with native executives, foreign members in the TMT have different advantages regarding knowledge, skills, resources, networks, innovativeness, and inclusiveness [6]. The presence of foreign executives can significantly promote foreign market entry, international knowledge sourcing activities, overseas research and development (R&D), business model innovation, corporate social responsibility, and firm performance [4, 7, 8]. However, the influence of foreign executives on firms’ innovation performance is still unknown.

Fierce market competition requires firms to have stronger innovation ability and outcomes [9]. Innovation refers to the utilization of a new technology to change an original production or operational process, produce new products, or deliver new services [10]. It is an important channel for firms to cultivate and maintain market competitiveness and is also a strategic activity involving high risk, high investment, and long-term commitment [11]. The executives are the decision-making and implementation group for firms’ strategy [12]; their attitude and leadership toward risk can largely determine the level of firms’ innovation input and output [11]. Scholars have indicated that foreign executives tend to exhibit a relatively broader vision, a higher risk appetite, and more interest in novelties, resulting in development of firms’ innovation projects [3]. Therefore, from the perspective of executives’ foreignness, our study examines the factors that influence innovation performance.

Managers’ personal preferences and experiences affect innovation input and output [13], but how executives can effectively promote innovation performance needs further exploration. Economic and societal development relies heavily on the pivotal role of digital technologies that can disrupt existing industries, innovates business models, and expand potential market opportunities [14]. Digital transformation can give firms more competitive power in fierce business environments by promoting resource integration and knowledge sharing, and strengthening innovative business models. It also increases customer satisfaction, enhances internal supervision and information transparency, improves corporate social responsibility and firm performance [15–18]. Presumably, digital transformation can be regarded as an effective tool for executives to improve innovation performance [19].

Previous literature has proven the crucial role of executives in firm’s digital transformation [20]. Executives’ motivation, commitment, managerial leadership, coordination abilities, and skills in recombining resources and seizing technological opportunities can effectively promote transformation [21]. However, digital transformation is typically accompanied by high investment, high risk, long cycles, and uncertain results, and executives often lack the motivation to meet these challenges [22–24]. This study investigates the relationship between foreign executives, digital transformation, and innovation performance.

This study is based on the upper echelons theory, and uses data from Chinese firms listed on the Shanghai and Shenzhen stock exchanges from 2011 to 2021. It is hypothesized that foreign executives improve innovation performance through digital transformation. Further analysis indicates that foreign executives promote radical rather than incremental innovation. Meanwhile, negative performance feedback and financing constraints are introduced as firms’ heterogeneous factors. Negative performance feedback enhances the impact of foreign executives on innovation performance, but financing constraints play a negative moderating role between foreign executives and innovation performance.

This study makes several contributions to the literature. First, it explains the role of foreign executives in relation to the innovation performance of firms, expanding the research on upper echelons theory. We extend the research of executives’ characteristics by arguing that foreign executives’ higher tolerance and advantages for innovation activities leads to the positive impact of foreignness in promoting long-term firm development. Second, this study sheds light on influencing factor of firm-level innovation from the dimension of executive nationality background. It accurately distinguishes the impact of foreign executives on dual innovation. In so doing, it also introduces the behavioral theory of firms and resource-dependence theory to reveal that performance pressure and resources are the constraints on the relationship between executives and innovation. Third, this study explores whether digital transformation can construct influence channels of foreign executives on innovation performance. It not only provides insights for firms to improve digital transformation by optimizing the TMT structure, but also proves the positive effect of digital transformation on innovation, which enriches the research on digital transformation.

## 2. Theoretical analysis and research hypothesis

### 2.1 Foreign executives and innovation performance

Upper echelons theory argues that executives make highly personalized strategy choices based on their cognitive abilities as well as values that are shaped by their life experiences and individual background [25, 26]. Thus, a firm’s behaviors and strategies are the results of executives’ characteristics and their risk inclination, which will directly influence firm’s performance and output [13, 27, 28]. The level of firms’ innovation performance is closely related to executives’ background, such as gender, age, educational background, career horizon, functional background, and working and life experiences [29–32]. TMTs’ richness of knowledge and skills and the channels for information and resources increase with the diversification of executives’ background and experiences, which contributes to firms’ innovation outcomes [33].

Nationality diversity is an important component of TMTs’ structure which has been proven to be a key role in the success or failure of innovation [7, 8, 34]. Foreign executives tend to be more proactive, motivated, resilient, and risk-tolerant in a foreign country than native managers working in their home country [35]. As overseas assignments that foreign executives choose voluntarily are uncertain and challenging, their choices are influenced by their cognitive and attitudinal characteristics of mobility and risk-taking [3, 36, 37]. Meanwhile, most foreign executives recruited by Chinese listed firms come from developed countries or regions. The social culture of these countries and regions emphasizes the value of individualism and self-determination—which can inculcate the ability for independent thinking and shape innovative personality characteristics such as creativity, uniqueness, and autonomy [38]. Executives who grow up in this social culture have more positive attitudes and a higher risk-taking tolerance, thus their firms also have a higher risk-taking tolerance [37]. However, China usually has a collectivist culture that emphasizes the pursuit of tradition and security in which individuals show strong obedience, consistency, and interdependence. This can suppress the growth of self-expression and creativity [39]. The business models chosen by Chinese executives are more robust, and their firms tend to avoid risky strategies and organizational change [40].

In addition to psychological and socio-cultural factors that lead foreign executives to have a higher tolerance for risk and innovation, their professional and management expertise instills confidence in their ability to handle risks and foster innovation [1]. Foreign executives hired by local firms are usually proven managerial talents with successful management experience and capabilities in the international market [2]. These executives’ past successes have endowed them with a profound understanding of the opportunities inherent in innovation [33]. This understanding enables them to delve deeper into avoiding potential pitfalls, thus reducing anxiety and self-doubt when confronting uncertain strategies [38]. Leveraging their extensive experience, broader knowledge base and cognitive abilities, they can accurately evaluate the various factors, potential risks, and expected returns involved in executing innovation strategies [3]. Compared to local managers, foreign executives also have a more extensive and sophisticated international network, providing additional knowledge, information, funding, technology, and channel support for innovation activities, ultimately enhancing the success rate of innovative strategies [5].

Foreign members also contribute to promote TMTs’ independence, which can help improve corporate governance and reduce risk aversion [41]. Most foreign executives are from developed countries with relatively well-honed corporate governance systems. Stronger contractual and legal concepts sensitize them to the rules and influence their value judgments, as reflected in management decisions and interactions with other executives [42]. Such values incline foreign executives to follow the relevant legal system and corporate governance norms in decision making [43]. Personal factors in the workplace have less influence on them. Therefore, their decision making is more objective and less opportunistic [42]. Simultaneously, it is difficult for foreign executives to integrate into local social circles and build new social relationship networks quickly, reducing the possibility of an executive conspiracy for personal interests [43]. Given foreign members’ high-quality internal attributes, TMTs tend to implement strategies conducive to firms’ long-term interests, developing innovation strategies and achieving better innovation performance.

Foreign executives enhance TMT heterogeneity, thereby fostering innovation activities. Prior research has shown that TMT heterogeneity broadens network channels, facilitating access to additional knowledge, information, and resources [33, 44]. Innovation is a distributed and open process, involving multiple participants, and the role of global networks in driving it is pivotal [45]. Foreign executives introduce globalized network channels to the organization, opening up international consulting opportunities, market intelligence, and innovation support resources [46]. These channels enable firms to generate more creative ideas through external connections and exchanges, while also mitigating innovation risks by fostering partnerships with overseas businesses [47]. As a result, TMT becomes more inclined to allocate more resources towards innovation, ultimately leading to more innovative outcomes [48]. Innovation emerges from the amalgamation of novel and existing elements, contingent upon the number diversity of available elements that can be combined [49]. The network channels of foreign executives enrich the resource pool of firms, significantly increasing the types of resource elements available. This expansion augments the chances of discovering fresh resource combinations, thereby enhancing the likelihood of generating groundbreaking ideas and products [50]. Moreover, some foreign executives, often hailing from technologically progressive firms or nations, contribute technological advantages to firms. They provide invaluable information and resources on advanced technologies, offering valuable insights for R&D projects [34]. This can assist firms in overcoming innovation obstacles and attaining superior outcomes.

Further, foreign executives improve TMT’s awareness and ability to respond to R&D activities, thereby boosting organizations’ innovation investment and performance [51]. Nationally diverse TMTs can achieve better performance in grappling with creative missions such as innovation [6, 52]. Foreign executives introduce fresh knowledge, skills, and viewpoints to the TMT, which, when combined with the insights of local executives, create favorable conditions for novel ideas and subsequent innovation projects [53]. Diversity in executives’ nationalities increases firms’ information-processing capacity, provides different perspectives on solutions to problems, reduces personal bias or groupthink, and improves strategies’ success rate [5]. Additionally, it enhances the firm’s knowledge absorption capacity, positioning it favorably for innovation and competitive edge in a rapidly evolving market. Consequently, this enables TMT to take a more proactive stance in investing in R&D endeavors, ultimately yielding superior innovation outcomes [54]. Thus, we propose the following hypothesis:

**Hypothesis 1 (H1): Foreign executives are positively related to innovation performance.**

### 2.2 Foreign executives and digital transformation

Digital technology is defined as the organic integration and cooperative control of information technology, computer technology, Internet technology, and other interaction and connection technologies [55]. Digital transformation uses various digital technology to update firms’ operational processes, managerial efficiency, business opportunities and models, and customer services and experiences [56]. Firms can employ advanced digital technology to comprehensively promote innovation in their organization and its capabilities, products, marketing, and service model to improve its production and business models [57]. Digital transformation can promote the information flow, enhance information-processing capabilities, optimize various internal processes, and change an organization’s culture and employees’ mindsets. This leads to further improvements in the business model, management transparency, corporate governance, market competitiveness, and innovations in technology and management [17, 58]. Therefore, digital transformation refers to the process through which companies accomplish organizational transformation and disruptive change to adapt to the digital economy [59].

The process involving organizational change and reengineering is long-term, yields uncertain results, and requires enormous resource inputs [60]. Owing to a weak digital foundation and low employee knowledge, traditional firms find it difficult to adapt quickly to digital technology. Digital native firms with strong technical capabilities and revolutionary business models occupy market-leading positions through cross-industry competition, constantly compressing traditional firms’ survival and development space [22]. This phenomenon forces traditional firms to rush into digital transformation and aggravates their high digital transformation failure rate. Digital transformation inherently requires substantial investment, long-term commitment, wide-range change, and high risk-tolerance, all of which are challenges for executives [24]. Principal-agent theory suggests that managers are prone to approach risky strategies with a negative outlook [11] and are also resistant to digital transformation [49]. However, a foreign TMT member can moderate such risk aversion and accelerate digital transformation. Most of the foreign executives hired by local firms are talent that have proved their management ability abroad and have successful management experience [61]. Existing research argues that based on foreign executives’ confidence obtained from a successful management career, they tend to have higher self-evaluations and less negative conceptions of uncertainty [62, 63]. Experienced executives often trust their experiences and abilities to handle risk and underestimate the complexity and difficulty of unpracticed strategies [47, 64]. They are confident in their ability to choose more daring strategic decisions [65]. Therefore, the lower risk perception of foreign executives may reduce managers’ resistance to digital transformation which involves substantial risk and uncertainty.

Foreign executives not only increase firms’ risk-taking preference but also enhances capacity to implement digital transformation. Digital transformation has triggered massive resource consumption and large-scale organizational reconfiguration, which impose higher demands on executives’ ability to gather and utilize resources [66]. Foreign executives have international social networks and resource channels far superior to those of local members. By integrating and utilizing scarce overseas resources, they can enhance the resource level of firms and provide them with the extensive resources required for digital transformation [67]. Additionally, when attracting executives with high salaries, firms tend to hire foreign executives with successful management experience and strong professional abilities [61]. Such executives have advanced management methods and decision-making experience, with a deep understanding of business models and philosophies [6, 52]. They can accurately grasp the development needs of digital transformation and properly deal with obstacles and problems in the process, thereby effectively promoting the implementation of digital transformation [1]. Therefore, introducing foreign executives can help firms enhance risk-bearing willingness and capacity, quickly establish mature overseas resource channels and advanced management systems. It also offers convenient financing conditions in the international capital market and high-quality cutting-edge information in the industry [3, 5]. Thus, we propose the following hypothesis:

**Hypothesis 2 (H2): Foreign executives are positively related to firms’ digital transformation.**

### 2.3 The mediating effect of digital transformation

As per the preceding argument, foreign executives tend to increase firms’ innovation performance because of their attitude toward risk, their independence in decision making, and the enhanced heterogeneity of the TMT that they represent. However, there is a need for deeper examination of the methods through which they can effectively encourage innovation performance. As the digital economy advances, scholars are increasingly focusing on how firms implement digital transformation to bolster their innovation performance [68]. Modern firm innovation requires the deep integration of traditional and digital technologies, as digital transformation is a breakthrough in innovation [69]. The multidimensional digitization of firms enhances their overall innovation capability and performance by optimizing businesses’ operations, production, and personnel [70], which can also generate organizational changes conducive to stimulating innovation projects [71]. Based on prior studies, our contention is that digital transformation can be a fruitful approach for foreign executives to foster innovation performance from the perspectives of cost reduction, consumer participation, and absorptive capacity improvement [72, 73].

Digital technologies tend to promote innovation by reducing the transaction, agency, and operation costs of innovation activities [68]. The digital era improves the information transparency and dissemination efficiency of firms’ external environment. It also makes the information and resources needed for innovation more accessible, reducing the transaction costs in innovation activities [74, 75]. Through digital transformation, internal information asymmetry can be alleviated, resulting in better control of innovation projects [76]. The application of digital technologies can transform and upgrade firms’ traditional innovation platforms and processes, lower R&D costs, thereby improve the success rate of innovation activities [77].

Digital transformation shortens the distance from firms to consumers [78]. Firms can quickly and accurately identify their users’ dynamic needs by using digital technologies proactively [79]. In this way, firms’ new products and services can accurately meet consumers’ requirements and find acceptance in the market [80]. The construction of a digital platform empowers consumers to directly access firms’ innovation activities and participate in the innovation process. This improves firms’ innovation performance by basing decisions relating to new product development on consumers’ needs [81]. Moreover, with the support of digital tools, foreign executives can understand domestic market needs and track changes in consumer demand more quickly and accurately, thus achieving rapid product iteration and continuous innovation success [75].

Digital transformation also provides an important pathway for promoting innovation outcomes through enhancing absorptive capacity. This refers to a firm’s ability to identify, introduce, and leverage new knowledge [68, 82]. Owing to the limitations and scarcity of firms’ internal information and resources, they need to have a strong absorptive capacity to obtain external information and resources for innovation activities [83]. The digital technology expands organizational boundaries and expedites the exchange of knowledge by empowering firms to overcome limitations of time and distance. It can also quickly gather globally available information and resources [74, 76]. Foreign executives can fully leverage digital technology to strengthen their firms’ absorptive capacity, reduce the uncertainty inherent in innovation, and improve innovation performance [68, 80]. Thus, foreign executives may promote innovation performance through implementing digital transformation. Thus, we propose the following hypothesis:

**Hypothesis 3 (H3): Digital transformation mediates the positive impact of foreign executives on innovation performance.**

## 3. Research design

### 3.1 Data and sample

China’s digital economy developed rapidly after 2010, and the availability of patent data for Chinese-listed firms was limited to the year 2021. Meanwhile, listed firms demonstrate a greater capacity and willingness to hire foreign executives compared with non-listed firms. Therefore, the sample for this study consisted of a panel dataset comprising Chinese firms listed on the Shanghai and Shenzhen stock exchanges during the period 2011 to 2021. Executives’ information and firm-level data were collected from the China Stock Market and Accounting Research Database as well as from the databases of the Shanghai and Shenzhen stock exchanges. Financial and insurance firms were excluded from the study owing to their uniqueness of financial data and insensitivity to innovation. Executives with a tenure of less than 1 year were also eliminated from samples as their limited time in the position would be difficult to affect firms’ long-term strategy. Furthermore, listed firms under special treatment were excluded from the study for mitigating the influence of extreme values. Finally, we excluded samples with incomplete information and data on key variables and obtained valid samples consisting of 14,411 observations. To mitigate the impact of outliers, we performed winsorization on all continuous variables at their 1st and 99th percentiles.

### 3.2 Variables

#### 3.2.1 Dependent variable

Our dependent variable was the firm’s innovation performance. We measured it as the number of firms’ annual patent applications which has been widely adopted in the existing related literature [84, 85]. Thus, the number of patent applications (Patent) equals to the natural logarithm of 1 plus annual patent applications.

#### 3.2.2 Independent variable

The independent variable was foreign executives (FE), defined as the ratio of foreign members in the TMT of listed firms [3]. Consistent with recent studies on TMTs, an executive is defined as a senior manager who can directly participate in a firm’s operations and management decisions, including membership of the TMT or executive board, or an individual with at least the rank of Vice President. Other position titles included were Chairman, Vice Chairman, Chief Executive Officer, Chief Financial Officer, Chief Technology Officer, Chief Operating Officer, Executive Vice President, and Senior Vice President [5, 8].

#### 3.2.3 Mediating variable

Firms’ degree of digital transformation (DT) was employed as the mediating variable. As in previous studies, digital transformation was measured as an index by creating a dictionary through text analysis of the listed firms’ annual reports [86, 87]. First, keywords related to digital transformation, such as artificial intelligence, blockchain, data management, multichannel, and digital infrastructure, were identified. On this basis, we built a dictionary of all variations in these keywords. Second, a machine learning algorithm was employed to calculate the frequency counts of these keywords from the annual reports of the firms in our sample. Finally, to protect the data from being skewed, we conducted logarithmic processing after adding 1 to the obtained data to achieve the overall index describing firms’ digital transformation.

#### 3.2.4 Control variables

We controlled for a range of factors that might affect firms’ innovation performance [68, 88]. These factors included firm size (FS), the liabilities-to-assets ratio (Lev), return on assets (ROA), total assets turnover (TAT), cash flow ratio (Cash), the shareholding rate of the top shareholder (Top), board size (BS), and board independence (BI). The year and industry were also controlled in this study. Table 1 presents a list of variables along with their corresponding explanations.

**Table 1:** Definition and measurement of variables.

Variable	Measurement method
Patent	Ln (Annual patent applications + 1)
FE	Number of foreign executives in TMT / TMT size
DT	Ln (the frequency of specific keywords related to digital transformation in firms’ annual reports + 1)
FS	Ln (total assets)
Lev	Total liabilities / total assets
ROA	Net profit / total assets
TAT	Operating income / total assets
Cash	Operating cash flow / total assets
Top	Share-holdings of top shareholder / total shares
BS	Ln (number of formal members of the board of directors)
BI	The number of independent directors / board size

### 3.3 Method

Firms are free to hire foreign executives, but only some hire them for strategic needs, which inevitably leads to self-selection bias in the samples. Therefore, this study employed the Heckman two-stage model to solve it. A probit model and an instrumental variable were used in the first stage to calculate the inverse Mills ratio (IMR) and the probability of firms hiring foreign executives. The dependent variable is a dummy variable measured as whether there was at least one foreign executive in TMTs (FE_Dum). According to previous studies, the instrumental variable is the number of years since the first foreign talent introduction policy was issued in the province where the listed firm is located (Policy) [42]. It is calculated as 1 plus the difference between the sample year and the year in which the province where the sample firm is located first issued the policy on the introduction of foreign talents. We expected this instrumental variable to be related to the probability of firm hiring foreign executives, but not influence digital transformation and innovation performance of the firm, thus meeting the Heckman’s two-stage model. All control variables were included as decision variables to calculate the probability of listed firms choosing foreign executives. The probit model (1) was constructed as follows.


$${FE\_Dum}_{t}=\alpha_{0}+\alpha_{1}{Policy}_{t}+\sum \alpha \;{Controls}_{t}+\varepsilon$$ (1)

Here, t, α, and ε represent the year, the coefficients of each variable, and the random disturbance, respectively. FE_Dum is the dependent variable, Policy is the instrumental variable, and Controls indicates all the control variables.

In the second stage, the regression models controlled for year and industry fixed effects were tested by adding the IMR obtained in the first stage as a new control variable. If the coefficient of IMR is significant, it shows that the bias caused by the sample’s self-selection has been effectively adjusted. We evaluated the association between foreign executives, firms’ digital transformation, and innovation performance by the following models. Models (2) and (3) were constructed to analyze the influence of foreign executives on firms’ innovation performance and digital transformation; and Model (4) tested digital transformation’s mediating role between foreign executives and innovation performance by using the causal steps approach.


$${Patent}_{t+1}=\alpha_{0}+\alpha_{1}{FE}_{t}+\alpha_{2}IMR+\sum \alpha \;{Controls}_{t}+\varepsilon$$ (2)

$${DT}_{t}=\alpha_{0}+\alpha_{1}{FE}_{t}+\alpha_{2}IMR+\sum \alpha \;{Controls}_{t}+\varepsilon$$ (3)

$${Patent}_{t+1}=\alpha_{0}+\alpha_{1}{FE}_{t}+\alpha_{2}{DT}_{t}+\alpha_{3}IMR+\sum \alpha \;{Controls}_{t}+\varepsilon$$ (4)

Here, t, α, and ε also represent the year, the coefficients of each variable, and the random disturbance, respectively. Patent is the dependent variable, representing innovative performance. DT is the mediating variable, representing digital transformation. FE is the independent variable, representing the ratio of foreign executives in TMT. IMR is used as a control variable. Additionally, Controls indicates all the other control variables. Previous studies indicate that there is a time delay between strategic decisions made by executives and the subsequent outputs [89]. Therefore, the foreign executives’ impact on innovation performance also exhibits a notable time lag. We adopt a one-year lag to treat the independent, mediating, and control variables.

## 4. Empirical results

### 4.1 Descriptive statistics

Table 2 presents the descriptive statistics for the main variables. The mean value of the patent applications index was 2.860, the medium value was 3.090. The range of this variable is from 0 to 7.180, and the variance reached 1.800. This indicates that some Chinese-listed firms have achieved remarkable results in innovation performance, but the number of innovation outcomes varies significantly among sample firms. Only 1% of the sample firms employed foreign executives, indicating that China’s listed firms still have few foreign executives in their TMTs. The digital transformation index had an average value of 1.980, a median of 1.790, a minimum value of 0.690, and a maximum value of 6.050. These figures suggest that nearly all Chinese-listed firms have appreciated the importance of digital technology and initiated their journey toward digital transformation.

**Table 2:** Descriptive statistics.

Variable	Obs.	Mean	Medium	S.D.	Min	Max
Patent	14411	2.860	3.090	1.800	0.000	7.180
FE	14411	0.010	0.000	0.050	0.000	1.000
DT	14411	1.980	1.790	1.120	0.690	6.050
FS	14411	22.190	22.020	1.300	19.520	26.400
Lev	14411	0.410	0.400	0.200	0.030	0.990
ROA	14411	0.040	0.040	0.070	-0.410	0.240
TAT	14411	0.670	0.560	0.460	0.050	2.910
Cash	14411	0.040	0.040	0.070	-0.210	0.260
Top	14411	0.340	0.320	0.150	0.080	0.760
BS	14411	2.120	2.200	0.200	1.610	2.710
BI	14411	0.380	0.360	0.050	0.300	0.600

Table 3 presents the results of the Pearson’s test and variance inflation factors (VIF) of the main variables. The absolute values of the correlation coefficients between all variables were less than the critical value of 0.600. The highest observed VIF value among all variables was 1.87, lower than the threshold value 10, indicating very limited multicollinearity in this study.

**Table 3:** Correlation matrix.

	1	2	3	4	5	6	7	8	9	10	11	VIF
1.Patent	1.000											
2.FE	0.051***	1.000										1.01
3.DT	0.091***	0.031***	1.000									1.04
4.FS	0.275***	-0.024***	-0.030***	1.000								1.68
5.Lev	0.068***	-0.072***	-0.089***	0.551***	1.000							1.88
6.ROA	0.087***	0.057***	0.000	-0.044***	-0.346***	1.000						1.38
7.TAT	0.017**	-0.003	0.027***	0.073***	0.199***	0.153***	1.000					1.13
8.Cash	0.059***	0.056***	-0.030***	0.061***	-0.145***	0.349***	0.092***	1.000				1.17
9.Top	-0.001	-0.026***	-0.134***	0.163***	0.037***	0.147***	0.094***	0.106***	1.000			1.08
10.BS	0.050***	-0.028***	-0.054***	0.268***	0.144***	0.022***	0.029***	0.048***	0.014*	1.000		1.63
11.BI	0.011	0.003	0.040***	-0.016*	-0.020**	-0.021**	-0.031***	-0.002	0.040***	-0.563***	1.000	1.51

Note

*, **, and *** indicate statistical significance at the 10%, 5%, and 1% levels, respectively.

### 4.2 Benchmark regression

Table 4 shows the regression results of the Heckman two-stage analyses. As shown in Column (1) of Table 4, the instrumental variable Policy was significant (β = -0.020, p < 0.01), and all IMRs were significant in columns (2)–(4). It indicates that the endogeneity problem caused by sample self-selection can be corrected using the Heckman two-stage regression method. As shown in column (2) of Table 4, foreign executives had a positive impact on patent applications (β = 0.540, p < 0.1). Therefore, foreign executives increase firms’ innovation performance, supporting Hypothesis 1. The results in Column (3) demonstrate that the association between foreign executives and digital transformation is significant (β = 0.352, p < 0.01). It indicates that foreign executives tend to enhance firms’ digital transformation, supporting Hypothesis 2. Column (4) shows that the correlation coefficient between the proportion of foreign executives and patent applications is significant (β = 0.470, p < 0.1), as is the correlation coefficient between digital transformation and patent applications (β = 0.198, p < 0.01). The results demonstrate that a firm’s digital transformation plays a mediating role between foreign executives and innovation performance. Foreign executives can promote firms’ innovation performance through digital transformation, supporting Hypothesis 3.

**Table 4:** Regression analysis results.

Variables	(1)	(2)	(3)	(4)
	FE_D	Patent_t+1_	DT	Patent_t+1_
Policy	-0.020***			
	(-3.720)			
FE		0.540**	0.352**	0.470*
		(2.222)	(2.426)	(1.957)
DT				0.198***
				(17.018)
FS	0.092***	0.755***	0.144***	0.727***
	(5.674)	(34.587)	(9.739)	(33.511)
Lev	-0.615***	-1.218***	-0.782***	-1.063***
	(-5.392)	(-8.284)	(-7.887)	(-7.284)
ROA	0.411	3.218***	0.228	3.173***
	(1.285)	(13.959)	(1.333)	(13.980)
TAT	0.001	0.151***	0.152***	0.121***
	(0.025)	(4.654)	(7.008)	(3.779)
Cash	0.736***	0.699***	-0.347**	0.768***
	(2.644)	(2.895)	(-2.020)	(3.200)
Top	-0.898***	-2.013***	-1.055***	-1.804***
	(-7.025)	(-10.380)	(-7.998)	(-9.390)
BS	-0.194*	-0.290***	-0.241***	-0.242***
	(-1.923)	(-3.303)	(-4.007)	(-2.783)
BI	-0.414	-0.853***	0.071	-0.867***
	(-1.141)	(-3.052)	(0.359)	(-3.134)
IMR		2.310***	0.755***	2.160***
		(10.122)	(4.849)	(9.547)
Constant	-2.262***	-17.913***	-2.645***	-17.389***
	(-5.259)	(-25.676)	(-5.584)	(-25.176)
Industry	Yes	Yes	Yes	Yes
Year	Yes	Yes	Yes	Yes
R^2^	0.045	0.423	0.266	0.434
N	14411	14411	14411	14411

Note: Robust standard errors are in parentheses.

*, **, and *** indicate statistical significance at the 10%, 5%, and 1% levels, respectively.

### 4.3 Robustness tests

The endogenous decision-making process regarding the recruitment of foreign executives has the potential to impact the analysis results. To alleviate concerns about sample-selection bias, we employed the propensity score matching (PSM) approach. All samples were divided into two subsamples based on foreign executives’ presence in a given year. We estimate the propensity scores for whether a firm recruits foreign executives using logit regression. The dependent variable is a dummy variable, which equals one if there is at least one foreign executive in the TMT, and zero otherwise. We utilized a 1:4 neighbor-matching method and took all control variables as covariates. The equalization test indicates that the average treatment effect on the treated (ATT) is statistically significant at the 1% level. Panel A of Table 5 shows that the covariates for treatment and control groups are not significantly different from each other, and therefore the matching criteria is met. Furthermore, we reran baseline regression using the data of treated and matched samples. The results presented in Panel B of Table 5 are consistent with the result of baseline regression, indicating that our analysis is robust.

**Table 5:** Robustness test: Propensity score matching analysis.

**Panel A: Propensity score matching results**							
**Variables**	**Treated**		**Control**		**Differences**		**T-statistics**
FS	22.135		22.143		-0.008		-0.13
Lev	0.366		0.368		-0.002		-0.31
ROA	0.054		0.053		0.001		0.14
TAT	0.660		0.656		0.004		0.22
Cash	0.055		0.055		0.000		-0.21
Top	0.305		0.302		0.003		0.42
BS	2.110		2.112		-0.002		-0.80
BI	0.377		0.377		0.000		0.22
**Panel B: Regression based on propensity score matching**							
**Variables**		**(1)**		**(2)**		**(3)**	
		**Patent**_**t+1**_		**DT**		**Patent**_**t+1**_	
FE		0.170***		0.081**		0.153***	
		(3.323)		(2.173)		(3.021)	
DT						0.209***	
						(10.068)	
FS		0.550***		0.094***		0.530***	
		(23.175)		(5.678)		(22.589)	
Lev		0.310*		-0.536***		0.422***	
		(1.883)		(-4.698)		(2.597)	
ROA		2.142***		-0.473		2.241***	
		(5.580)		(-1.593)		(5.950)	
TAT		0.165**		0.248***		0.113*	
		(2.393)		(5.322)		(1.671)	
Cash		-0.811**		-1.100***		-0.580	
		(-2.065)		(-4.137)		(-1.485)	
Top		-0.253		-0.487***		-0.151	
		(-1.506)		(-4.373)		(-0.911)	
BS		0.300**		-0.082		0.317**	
		(2.015)		(-0.755)		(2.156)	
BI		-0.075		0.603*		-0.201	
		(-0.149)		(1.652)		(-0.406)	
Constant		0.170***		-0.993**		-11.351***	
		(3.323)		(-2.383)		(-19.060)	
Industry		Yes		Yes		Yes	
Year		Yes		Yes		Yes	
R^2^		0.401		0.264		0.415	
N		4236		4236		4236	

Note: Robust standard errors are in parentheses.

*, **, and *** indicate statistical significance at the 10%, 5%, and 1% levels, respectively.

Owing to the sustainability and long periodicity of innovation projects, foreign executives may require a longer duration to exert their influence on innovation output. Thus, we introduced two- and three-year lags to the independent variable before proceeding with the second stage of the Heckman regression analysis. Columns (1) and (2) of Table 6 show the test results of two-year lagged effects. Columns (3) and (4) demonstrate the test results of three-year lagged effects. All the test results are the same as with baseline regression, indicating that our results are still robust after the independent variables lag for a longer period.

**Table 6:** Robustness test: Additional lagged effects.

Variables	(1)	(2)	(3)	(4)
	Patent_t+2_	Patent_t+2_	Patent_t+3_	Patent_t+3_
FE	0.819***	0.787***	0.744**	0.747**
	(2.856)	(2.762)	(2.125)	(2.159)
DT		0.173***		0.147***
		(12.358)		(8.901)
FS	0.729***	0.706***	0.732***	0.714***
	(27.043)	(26.362)	(23.713)	(23.242)
Lev	-1.099***	-0.974***	-1.131***	-1.031***
	(-6.103)	(-5.446)	(-5.521)	(-5.057)
ROA	3.640***	3.606***	4.179***	4.107***
	(11.980)	(11.961)	(10.557)	(10.402)
TAT	0.155***	0.133***	0.176***	0.161***
	(4.043)	(3.515)	(4.074)	(3.753)
Cash	0.540*	0.626**	0.742**	0.829**
	(1.829)	(2.132)	(2.223)	(2.495)
Top	-1.989***	-1.819***	-2.084***	-1.951***
	(-8.391)	(-7.745)	(-7.768)	(-7.327)
BS	-0.268**	-0.243**	-0.341***	-0.322***
	(-2.543)	(-2.327)	(-2.853)	(-2.715)
BI	-1.041***	-1.078***	-1.056***	-1.088***
	(-3.107)	(-3.248)	(-2.752)	(-2.858)
IMR	2.177***	2.066***	2.274***	2.195***
	(7.734)	(7.398)	(7.137)	(6.934)
Constant	-16.884***	-16.479***	-17.006***	-16.722***
	(-19.696)	(-19.386)	(-17.492)	(-17.320)
Industry	Yes	Yes	Yes	Yes
Year	Yes	Yes	Yes	Yes
R^2^	0.415	0.423	0.404	0.411
N	10452	10452	8214	8214

Note: Robust standard errors are in parentheses.

*, **, and *** indicate statistical significance at the 10%, 5%, and 1% levels, respectively.

To exclude the influence of index selection and variable measurement on the results, we re-performed the regression analysis using alternative measures of digital transformation and innovation performance. First, by referring to previous literature, we chose the proportion of digitization related items in the year-end intangible asset details regarding the added value of total intangible assets as an alternate variable for firms’ digital transformation (DT_IA) [90]. Intangible assets related to the digitization were identified as the total intangible assets labeled with software, network, client, digital, intelligence, and management system. They were disclosed in the appendices of the financial reports of Chinese listed firms. Second, the measurement method of the dependent variable, innovation performance, was replaced by the annual number of patents granted (G-Patent) to listed firms [28]. It was calculated as the natural logarithm of the sum of 1 and the number of licensed patents. Then, the second stage of Heckman regression was employed. Column (1) of Table 7 reports the positive impact of foreign executives on firms’ intangible assets related to the digitization. Column (2) shows that the intangible assets related to digitization can also play a mediating role between foreign executives and innovation performance. Columns (3) and (4) show that after using granted patents to measure innovation performance, the regression results were consistent with our baseline analyses, supporting all hypotheses.

**Table 7:** Robustness test: Alternative measure for digital transformation and innovation performance.

Variables	(1)	(2)	(3)	(4)
	DT_IA_t+1_	Patent_t+1_	G-Patent_t+1_	G-Patent_t+1_
FE	0.591***	0.496*	0.631***	0.570**
	(9.799)	(1.940)	(2.793)	(2.555)
DT_IA		0.097*		
		(1.897)		
DT				0.173***
				(15.925)
FS	-0.010***	0.752***	0.725***	0.701***
	(-3.028)	(34.190)	(35.611)	(34.611)
Lev	-0.043*	-1.177***	-1.243***	-1.108***
	(-1.865)	(-7.953)	(-9.133)	(-8.209)
ROA	0.059	3.211***	2.175***	2.135***
	(1.283)	(13.709)	(10.404)	(10.397)
TAT	0.027***	0.145***	0.119***	0.093***
	(4.894)	(4.462)	(3.986)	(3.142)
Cash	-0.039	0.673***	0.949***	1.009***
	(-0.890)	(2.752)	(4.186)	(4.480)
Top	-0.088***	-2.003***	-1.927***	-1.745***
	(-2.869)	(-10.264)	(-10.628)	(-9.721)
BS	-0.027*	-0.286***	-0.348***	-0.307***
	(-1.880)	(-3.242)	(-4.274)	(-3.787)
BI	0.047	-0.852***	-0.986***	-0.998***
	(1.014)	(-3.031)	(-3.780)	(-3.859)
IMR	0.081**	2.286***	2.383***	2.253***
	(2.294)	(9.964)	(11.167)	(10.660)
Constant	0.133	-17.812***	-17.356***	-16.898***
	(1.219)	(-25.327)	(-26.543)	(-26.109)
Industry	Yes	Yes	Yes	Yes
Year	Yes	Yes	Yes	Yes
R^2^	0.220	0.419	0.434	0.444
N	14237	14237	14411	14411

Note: Robust standard errors are in parentheses.

*, **, and *** indicate statistical significance at the 10%, 5%, and 1% levels, respectively.

## 5. Further analyses

### 5.1 Heterogeneity of innovation performance

We also conducted several further analyses to confirm our assumptions regarding the mechanism through which foreign executives influence firms’ innovation performance. Dual innovation, which includes both incremental and radical innovation, was introduced to examine the impact of foreign TMT members on the heterogeneity of innovation performance [91]. Incremental innovation is defined as minor changes and modifications to firms’ incumbent products and technologies, indicating cumulative and continuous improvements from existing technologies [92, 93]. By contrast, radical innovation refers to major departures and discontinuous changes from existing technological routes and leapfrogging in completely new products and services [94]. Research has shown that overseas executives preferred invention patents, as opposed to utility model and design patents [28]. Thus, we compared the distinct impacts of foreign executives on the performance of incremental innovations and radical innovations.

The China National Intellectual Property Administration classifies patents into three types: invention patents, new utility patents, and new design patents. These are commonly used by scholars to analyze and measure organizations’ innovation performance [95]. Invention patents are typically awarded to new products or substantive inventions, which undergo longer review periods and stricter approval procedures than new utility and new design patents [96]. Therefore, following the recent literature, we measure firms’ radical innovation performance by employing the natural logarithm of 1 plus the number of invention patent applications [97]. The natural logarithm of 1 plus the number of utility and design patent applications was used to measure incremental innovation performance [98].

We replaced the dependent variable in Model (2) with radical innovation performance (R-Patent) and incremental innovation performance (I-Patent) separately. Columns (1) and (2) of Table 8 show the second stage of Heckman two-stage regression results of the association between foreign executives and dual innovation performance. The proportion of foreign executives was shown to have a positive impact on invention patent applications (β = 0.807, p < 0.01) but no influence on utility and design patent applications (β = 0.095, p > 0.1). The empirical results demonstrate that foreign executives are able to increase radical innovation performance but not incremental innovation performance. The improving effect of foreign executives on innovation performance is reflected in the enhancement of firms’ innovation ability and innovation quality.

**Table 8:** Further analysis.

Variables	(1)	(2)	(3)	(4)	(5)	(6)
	R-Patent_t+1_	I-Patent_t+1_	Patent_t+1_	Patent_t+1_	Patent_t+1_	Patent_t+1_
Sample	All		NPF	Non-NPF	High-KZ	Low-KZ
FE	0.847***	0.166	0.587**	0.532	0.040	1.011***
	(3.752)	(0.699)	(2.215)	(1.013)	(0.115)	(3.070)
FS	0.685***	0.690***	0.779***	0.685***	0.756***	0.713***
	(33.150)	(32.766)	(31.729)	(14.454)	(25.422)	(22.019)
Lev	-0.956***	-1.043***	-1.415***	-0.529	-1.425***	-0.554**
	(-6.979)	(-7.361)	(-8.585)	(-1.629)	(-6.986)	(-2.395)
ROA	2.484***	2.959***	3.464***	0.823	3.013***	3.491***
	(11.913)	(13.606)	(13.562)	(1.148)	(10.503)	(8.758)
TAT	0.103***	0.137***	0.173***	0.074	0.137***	0.171***
	(3.562)	(4.491)	(4.665)	(1.124)	(3.326)	(3.172)
Cash	0.331	1.020***	0.954***	0.584	1.353***	-0.656
	(1.484)	(4.430)	(3.438)	(1.157)	(4.068)	(-1.639)
Top	-1.626***	-1.929***	-2.154***	-1.704***	-1.557***	-2.452***
	(-8.857)	(-10.192)	(-9.771)	(-4.238)	(-5.674)	(-9.140)
BS	-0.158*	-0.421***	-0.320***	-0.161	-0.222*	-0.334**
	(-1.899)	(-5.037)	(-3.207)	(-0.892)	(-1.900)	(-2.550)
BI	-0.481*	-1.220***	-0.644**	-1.560***	-0.680*	-0.981**
	(-1.784)	(-4.548)	(-2.038)	(-2.661)	(-1.797)	(-2.399)
IMR	1.522***	2.557***	2.420***	2.103***	1.940***	2.621***
	(7.076)	(11.543)	(9.337)	(4.426)	(6.069)	(8.155)
Constant	-16.076***	-17.015***	-18.579***	-16.016***	-17.497***	-17.339***
	(-24.474)	(-25.210)	(-23.352)	(-11.093)	0.040	(-17.264)
Industry	Yes	Yes	Yes	Yes	Yes	Yes
Year	Yes	Yes	Yes	Yes	Yes	Yes
R^2^	0.366	0.403	0.427	0.426	0.439	0.415
N	14411	14411	10950	3461	7900	6511

Note: Robust standard errors are in parentheses.

*, **, and *** indicate statistical significance at the 10%, 5%, and 1% levels, respectively.

### 5.2 Heterogeneity of firms

To further investigate the impacts of firm heterogeneity factors on the relationship between foreign executives and innovation performance, we introduced two variables on firms’ performance and financing status. First, negative performance feedback may influence the development mechanism of foreign executives on innovation performance. Behavioral theory of the firm argues that unrealized performance levels drive firms to improve their performance or reverse their tendency toward decline by initiating search activities [99]. The disparity between performance and aspirations raises executives’ awareness of the seriousness of the firm’s problems, forcing them to improve their risk tolerance levels and make strategic adjustments [100]. Recent studies show that negative performance feedback can encourage firms to introduce new products and technologies, improve R&D intensity, and employ innovative strategies [101, 102]. Performance shortfalls are more likely to motivate foreign executives to respond quickly to a decline in their performance relative to their targets and promote R&D strategies to achieve better innovation output than usual [103].

Thus, we introduce the grouping variable negative performance feedback (NPF), measured as the difference between a firm’s aspiration ROA and actual ROA, based on previous related studies [99, 104]. Aspiration ROA is a weighted measure of social and historical aspirations. Social aspiration is the average ROA of all firms within the focal firm’s industry, excluding the focal firm. Historical aspiration is measured using a weighted average of firms’ past ROA. If the actual ROA exceeds the aspiration, NPF equals 0; if the actual ROA is below the aspiration level, NPF equals 1. We used model (2) to run grouping regression based on NPF values. Column (3) in Table 8 reports the association between foreign executives and patent applications in NPF sample; the coefficient of foreign executives was positive and significant (β = 0.519, p < 0.1). Column (4) shows that the coefficient of foreign executives was insignificant (β = 0.471, p > 0.1) for the non-NPF samples. The results indicate that the effect of foreign executives on innovation performance is stronger when a firm fails to achieve its expected performance.

Second, we explored the association between the presence of foreign executives and innovation performance in firms subject to different financing constraints, which are major problems for the further development of Chinese firms [105]. Recent studies have argued that financing constraints harm strategic decisions because they limit strategy development and organizational changes such as investment in fixed assets and inventory, R&D input, innovation activities, foreign trade, and the internationalization process [106–108]. Executives’ investment options and their amount of discretionary capital are restricted when firms face strict financing constraints [109]. Executives must set financing priorities for projects according to their strategic importance and ensure that limited resources are invested in basic business activities rather than in high-risk and high-cost innovation projects [110, 111]. Therefore, financing constraints may limit the significant contributions by foreign TMT members to innovation performance.

We introduce the grouping variable of financing constraints, measured as the KZ index, in line with previous research [105, 112]. The KZ index is calculated using five indicators: (i) the ratio of net cash flow from operations to total assets in the previous period, (ii) the ratio of cash dividends to total assets in the previous period, (iii) the ratio of cash holdings to total assets in the previous period, (iv) the gearing ratio, and (v) the Tobin’s Q. As the KZ index increases, the sample firm faces a higher level of financing constraints. We grouped the samples according to the median of KZ and reran the Model (2) regression. Column (5) of Table 8 reports the association between the proportion of foreign executives in TMT and patent applications in the high-KZ samples; the coefficient of foreign executives was insignificant (β = -0.060, p > 0.1). Column (6) shows that the coefficient of foreign executives was positive and significant (β = 0.990, p < 0.01) for the low-KZ samples, indicating that the influence of foreign executives on innovation performance is more significant in firms operating in a more favorable financing environment.

## 6. Discussion and conclusion

This study aligns research streams by investigating the connections among executives’ characteristics, digital strategy, and innovation output in an integrated model using Chinese-listed firms’ data. Our results reveal that foreign executives can significantly expedite digital transformation, consequently bolstering innovation performance of firms. To further validate the influence of foreign executives on innovation performance, we selected incremental and radical innovation performance as the variables that compose overall innovation performance. The results of the analysis indicate that foreign TMT members exert influence on driving radical innovation rather than incremental innovation. This can be clarified through the reasoning that foreign executives are better equipped to handle challenges and demonstrate greater commitment toward achieving high-quality innovation outcomes. Regarding the moderating effect, the empirical results demonstrate that foreign executives can promote innovation performance only in firms facing negative performance feedback and weaker financing constraints. This suggests that the operational pressures faced by the executives and the financing environment of the company are crucial for innovation output.

### 6.1 Theoretical contribution

Our study makes several incremental theoretical contributions to extant research. First, this study adds to existing literature on the influence of foreign TMT members on strategic decisions and firm-level outcomes, expanding the core tenet of upper echelons theory. From a socio-cultural and psychological perspective, foreign executives are more inclined to take risks and exhibit optimistic attitudes toward strategies with unpredictable outcomes, such as R&D and digital transformation [35, 37]. Based on corporate governance and a resource-based view, they also increase independence and heterogeneity of the TMT, contributing to improved decision quality and related information, resources, and technical advantages. From the perspective of digital transformation and innovation strategies, we complement an under-explored role of TMT internationalization in executives’ characteristics research. The results align with current research on the economic consequences of TMT internationalization, which affirms that foreign TMT members contribute positively toward long-term strategies and firm-level outcomes.

Second, this study opens the black box of influencing factors of innovation strategy by expanding the antecedents to executives’ foreignness that shapes their cognitive and management abilities. Most of the existing literature focuses on the impact of executives’ overseas experience on innovation input or output, and few studies have linked foreign executives with innovation strategy directly. Our study provides new evidence for the research on firm innovation management, emphasizing the introduction of foreign executives as a core driving factor in innovation activities. This finding has positive implications in the complex environment of the current economic downturn and fierce competition among firms that forces firms to constantly strengthen R&D projects to maintain long-term competitiveness. Meanwhile, we further reveal the differentiated impact of foreign executives on different types of innovation output, highlighting the advantageous contribution of foreign executives in radical innovation. Firms with all the advantages provided by foreign executives enhance their absorptive capacity and R&D confidence, resulting in preferring more difficult radical innovations. Additionally, performance aspirations and financing constraints are introduced to examine boundary conditions between executives’ foreignness and innovation performance. This study integrates the behavioral theory of firms and resource-dependence theory with innovation management research by indicating that firms with greater performance pressure and abundant resources can better conduct innovation activities.

Third, we broaden the research scope of antecedents and consequences of digital transformation. At present, digital transformation has become an urgent need for firms’ future development, and the role of digital transformation for firms has become one of the current research hotspots. However, literature that explores the influencing factors of digital transformation from the perspective of executives’ characteristics is scarce. Thus, we utilize an empirical research model to reveal the promoting role of executives’ nationality background in digital transformation. Simultaneously, we demonstrate that digital transformation can be an effective tool for executives to increase firms’ innovation performance. Digital transformation’s positive impact on innovation performance lays a solid theoretical foundation for firms to strengthen their innovation capabilities by employing digital technology.

### 6.2 Policy implications

This study also holds some practical implications. First, it provides a reference for selecting and employing executives. Boards should attach importance to foreign members in establishing or optimizing the TMT, especially when facing high-risk strategies such as digital transformation and innovation. Firms should strongly recognize the particularity of foreign executives and take full advantage of their experience, abilities, and resources geared toward coping with creative and complex activities.

Second, owing to the growing intensity of competition and profound influence of the digital economy, firms should increase their willingness to implement organizational changes and take risks by, for example, investing heavily in digital transformation and innovation activities [24]. Executives should fully integrate digital orientation and innovation strategy, leveraging digital technologies to enhance firms’ innovation capability and output. However, the implementation of digital transformation and innovation projects place a great demand on firms’ resources [66], which means that financing constraints are a serious obstacle. Therefore, firms should enhance their resource-gathering ability and crack financing bottlenecks to meet their development needs and strategic paths.

Third, the board of directors must objectively comprehend the decision-making tendencies exhibited by foreign executives during adverse situations encountered by the firm. When they faces operating conditions where performance expectations cannot be met, foreign executives can help firms focus limited resources on strategies that can quickly overcome difficulties, such as digital transformation, innovation activities. However, it is crucial to recognize that while these high-value strategies offer significant potential, they also carry substantial risks. Given their risk-taking tendencies and confidence in their own experience and risk-management abilities, foreign executives might underestimate the risks associated with digital transformation or innovation strategies, especially when under significant performance pressure. They may even engage in unethical behavior in order to quickly gain recognition and support from stakeholders, or to overturn negative stereotypes and prejudices held by local executives, thereby increasing the possibility of harming the company’s interests. Therefore, during operational lows, shareholders and the board have a dual responsibility. On the one hand, they should provide sufficient support to foreign executives, fully leveraging the diverse advantages brought by these executives in managing digital transformation and innovation strategies, thereby improving the efficiency and effectiveness of strategic decision-making. On the other hand, it is essential to strengthen supervision of foreign executives, ensuring that they do not adopt overly risky strategies or engage in unethical behavior. This balanced approach is crucial for safeguarding the interests of the company while leveraging the unique strengths and perspectives that foreign executives bring to the table.

### 6.3 Limitations and future research

This study also has limitations which can indicate the direction of future research. First, we used the numbers of patent applications and patents granted to describe firms’ innovation performance. In the current literature, patents represent an essential and popular measurement of innovation. However, not all innovation outcomes are patentable. Some firms may keep technological secrets and protect their innovation products by eschewing patents. Thus, patent applications can only partially capture a firm’s innovation performance, and future studies should consider alternative more precise variables. Second, this study only evaluated foreign member in a TMT from a single dimension, that is the ratio of foreign members in the TMT. We did not consider the comprehensive influence of multiple foreign executives and the heterogeneity of foreign executives. In future studies, more empirical analysis should be added to test the impact of nationality diversity of TMT on firm strategies. Third, the research subjects’ data were confined to foreign executives’ demographic background characteristics, which we used to infer their cognitive processes, behavioral logic, and decision-making preferences. However, we did not directly include or measure the psychological characteristics of foreign executives. In future studies, relevant theories and methods of psychology should be applied using interviews, experiments, questionnaires, and other methods for more detailed measurement and description of foreign executives’ mental activities. This will help open the black box of the influence of executives’ characteristics on firm strategies.

## Supporting information

S1 Data Regression data. (XLS)
